# Developing a core outcome set for people living with dementia at home in their neighbourhoods and communities: study protocol for use in the evaluation of non-pharmacological community-based health and social care interventions

**DOI:** 10.1186/s13063-018-2584-9

**Published:** 2018-04-24

**Authors:** Andrew J. E. Harding, Hazel Morbey, Faraz Ahmed, Carol Opdebeeck, Ying-Ying Wang, Paula Williamson, Caroline Swarbrick, Iracema Leroi, David Challis, Linda Davies, David Reeves, Fiona Holland, Mark Hann, Ingrid Hellström, Lars-Christer Hydén, Alistair Burns, John Keady, Siobhan Reilly

**Affiliations:** 1 Lancaster University, Lancaster, UK; 2 Manchester Metropolitan University, Manchester, UK; 3 University of Liverpool, Liverpool, UK; 4 University of Manchester, Manchester, UK; 5 Linköping University, Linköping, Sweden; 6 Greater Manchester Mental Health NHS Foundation Trust, Manchester, UK

**Keywords:** Core outcome set, Dementia, Non-pharmacological interventions, Community-based programmes, Neighbourhood, Delphi method, Public involvement

## Abstract

**Background:**

The key aim of the study is to establish an agreed standardised core outcome set (COS) for use when evaluating non-pharmacological health and social care interventions for people living at home with dementia.

**Methods/design:**

Drawing on the guidance and approaches of the Core Outcome Measures in Effectiveness Trials (COMET), this study uses a four-phase mixed-methods design:

1. Focus groups and interviews with key stakeholder groups (people living with dementia, care partners, relevant health and social care professionals, researchers and policymakers) and a review of the literature will be undertaken to build a long list of outcomes.
2. Two rounds of Delphi surveys will be used with key stakeholder groups. Statements for the Delphi surveys and participation processes will be developed and informed through substantial member involvement with people living with dementia and care partners. A consensus meeting will be convened with key participant groups to discuss the key findings and finalise the COS.
3. A systematic literature review will be undertaken to assess the properties of tools and instruments to assess components of the COS. Measurement properties, validity and reliability will be assessed using the Consensus-based Standards for the Selection of Health Measurement (COSMIN) and COMET guidance.
4. A stated preference survey will elicit the preferences of key stakeholders for the outcomes identified as important to measure in the COS.

**Discussion:**

To the best of our knowledge, this study is the first to use a modified Delphi process to involve people living with dementia as a participant group. Though the study is confined to collecting data in the United Kingdom, use of the COS by researchers will enhance the comparability of studies evaluating non-pharmacological and community-based interventions.

**Trial registration:**

The study is registered on the COMET initiative, registered in 2014 at comet-initiative.org.

**Electronic supplementary material:**

The online version of this article (10.1186/s13063-018-2584-9) contains supplementary material, which is available to authorized users.

## Background

It is currently estimated that there are 850,000 people living with dementia in the UK, two-thirds of whom live in their own homes. Being in a familiar neighbourhood and family surroundings can help people living with dementia cope better with their everyday lives and moreover, the majority of people living with dementia want to stay in their own home [1, 2].

One-third of people living with dementia live alone and are particularly reliant on support from family members, community services or home care agencies [1, 3]. In the UK, and in recent years, the range of non-pharmacological support available to people living with dementia to retain independence in their own homes and neighbourhoods has increased [3, 4]. Consequently, there is a growing demand for evidence-based care and interventions that seek to improve the outcomes for people living with dementia [5] with the corollary being the identification of outcomes that are deemed important by key stakeholders.

In recent years, a limited consensus has been reached about what outcomes should be measured in dementia services and studies. For example, in 2007, the International Psychogeriatric Association published a consensus statement calling for clear predefined outcome measures when assessing treatment benefits for dementia. The association recommended that outcomes could include: the effect of interventions on people living with dementia’s cognition, behavioural and psychological symptoms; quality of life; global assessments and activities of daily living. In addition, it was recommended that outcomes could encompass the effects on care partners [6].

With a focus on disease modification interventions in people living with mild to moderate dementia, Webster and colleagues [7] recommended that cognition (measured with the cognitive subscale of the Alzheimer’s Disease Assessment Scale [8] or the Mini Mental State Exam [9]) and biological markers (magnetic resonance imaging [10]) should be the only core outcome domains. Other recommendations were made for important, but non-core, domains and included activities of daily living (Disability Assessment for Dementia [11]), global functioning (Clinical Dementia Rating [12]), neuropsychiatric aspects (Neuropsychiatric Inventory [13]) and quality of life (Dementia Quality of Life [14]).

Another study identified outcomes suitable when examining the effect of psychosocial interventions in dementia care. Using a consensus meeting approach, this study identified 22 measures across nine conceptual domains covering patient-based measures (mood, quality of life, activities of daily living, instrumental activities of daily living and behaviour), carer-based measures (mood, quality of life and burden) and staff-based measures (morale) [15].

Currently, the only ‘effective practice and health systems’ review registered with the Cochrane Dementia and Cognitive Improvement Group is of case management for people living with dementia [16]. The researchers in this review used the following categorisation for synthesising outcomes: avoidance of institutionalisation; numbers of admissions; quality of life/wellbeing; cognitive functioning; neuropsychiatric/behavioural and psychological symptoms; mood; activities of daily living; and social engagement. This review and many completed and ongoing Cochrane reviews of interventions for people living with dementia face a high degree of variation in outcome measures. Limited consistency between studies can lead to marked heterogeneity and reporting biases [16, 17], thus impeding comparison of findings across studies and making meta-analyses and interpretation of results difficult [18].

Furthermore, existing measures may not detect, or include, outcomes that are important and meaningful to people with dementia, whose perspectives are often not represented [19, 20]. More generally, although recent evidence suggests patient or public involvement in identifying priorities and outcomes of importance is still an emerging area [20], it is often not done [7, 15] or implemented poorly [21]. Yet, studies that have done so have identified outcomes that were not previously identified by clinicians [22]. Given that nearly two-thirds of people with dementia live at home, outcome measures in existing studies do not necessarily reflect the types of outcomes these people seek from dementia care in neighbourhood and community settings [23].

This study is a dedicated work programme (work programme 3) of the Economic and Social Research Council (ESRC) and National Institute for Health Research (NIHR) Neighbourhoods and Dementia mixed-methods research study (http://www.neighbourhoodsanddementia.org; henceforward, the Neighbourhoods and Dementia study). The five-year Neighbourhoods and Dementia study (2014–2019) is one of the studies being funded under key commitment 12 of the first Prime Minister’s Challenge on dementia, a commitment of funding for social science research, and explores the meanings, experiences and composition of neighbourhoods for people living with dementia, their care partners and families, and other groups and individuals with whom they have contact [24].

Led by Professor John Keady (chief investigator) at the Division of Nursing, Midwifery and Social Work (School of Health Sciences, University of Manchester), the Neighbourhoods and Dementia programme is framed around people, spaces and places and aims to:

- Address the meanings, experiences and structure of neighbourhoods for people living with dementia, their care partners and other in-contact-groups and individuals
- Learn from the process and praxis of making people living with dementia and their care partners core to the research agenda
- Encourage innovative technological advances in dementia studies and in the development of a neighbourhood model of dementia
- Build capacity within the research community and the networks of people living with dementia and their care partners
- Develop the evidence base, methods and measures for understanding the significance of neighbourhoods for people living with dementia and their care partners
- Create, test and evaluate interventions that are pertinent to a neighbourhood model of dementia

The central aim of work programme 3, led by Dr Siobhan Reilly at the Division of Health Research (Faculty of Health & Medicine, Lancaster University) of the Neighbourhoods and Dementia study is to develop a core outcome set (COS) that can be used when evaluating non-pharmacological health and social care community-based interventions for people living with dementia at home and in their own neighbourhood locality. As such, the emphasis is on identifying outcomes that are important to the person living with dementia.

The study will:

i) Identify and attain a consensus around which outcomes should be measured from the perspective of key stakeholders (people living at home with dementia, care partners, health and social care professionals, researchers and policymakers).
ii) Review and recommend how outcomes should be measured.

Throughout this study, the five key stakeholders groups are defined as:

1. *People living with dementia*: People either formally or self-diagnosed with dementia and who live at home.
2. *Care partners*^1^: People with current or past experiences of providing care for a person living with dementia.
3. *Health and social care professionals*: People currently or recently employed by a public or private organisation that provides care or support in a health or social setting for people living with dementia.
4. *Researchers*: People with current or recent experience of undertaking dementia-related research (i.e. as denoted by being a lead or co-author on dementia-related peer-reviewed publications or involvement in current dementia-related research).
5. *Policymakers*: People in a senior role with influence to shape national, regional or local dementia policy or who are able to commission dementia service provision. This includes those who plan services.

There are additional inclusion criteria, and these are outlined in the recruitment sections of the protocol.

## Methods/design

**Fig. 1:**
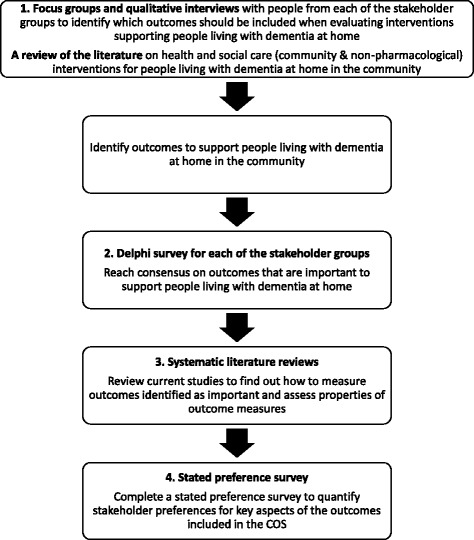
Study design of the development of a core outcome set for evaluating community health and social care interventions for people who live at home with dementia

There is no recognised gold standard for the development of a COS. This study applies an approach that uses guidance from the Core Outcome Measures in Effectiveness Trials (COMET) and includes a four-phase mixed-methods study design (Fig. 1).

### Phase 1: Identification of potential outcomes through qualitative data collection and a literature review

#### Phase 1.1: Interviews and focus groups

Note that it was necessary to complete phase 1 and the early stages of phase 2 to inform the design of the Delphi surveys. Thus, the sections on phase 1 and the member involvement undertaken in relation to the Delphi surveys in phase 2 describe what we have already done. However, no substantive findings are presented in this paper.

Focus groups and interviews were used to identify which outcomes are important for people living at home with dementia within each stakeholder group. Group size and specific approaches depended on the stakeholder group and approaches differed due to methodological and practical considerations, such as individual capacity and preferences, and time pressure of care professionals.

Focus groups have been found to be appropriate for research involving people living with dementia [19, 25, 26]. There are a number of advantages to group discussions. They can enhance the quality of interaction, reduce the pressure on individuals to respond, and provide mutual support and opportunities for shared experiences to stimulate the memories of people living with dementia [19].

In the study, people living with dementia who lacked mental capacity and who had an identified personal consultee were invited for an interview rather than a group discussion. This provided an important opportunity to enable the researcher to build rapport with the person living with dementia and to foster a good relationship [27].

#### Phase 1.1: Recruitment:

##### Recruitment of people living with dementia

We recruited a sample of people living with dementia who met the following criteria:

Inclusion criteria:

- They had a diagnosis of dementia (self or professional reported).
- They lived at home in the community in the north-west of England.
- They had capacity to understand and consent to participate in the study (including those who could consent in the moment), or had a personal consultee who was identified and approached if individuals living with dementia were not able to consent in the moment.
- They were able to converse in English.

Exclusion criteria:

- They were living in institutional care (nursing home, care home, or hospital).
- They were too unwell to participate.
- They did not speak English.
- They did not have capacity to consent to participate in the study and did not have a personal consultee, or where a personal consultee was identified but declined to give their agreement to approach the person living with dementia.

To identify people living with dementia, we liaised with relevant clinical research network staff at National Health Service (NHS) trusts and staff within third-sector organisations (e.g. Alzheimer’s Society and local memory cafes) to identify potential participants.

##### Recruitment process for people living with dementia

Potential participants were given information about the study and the opportunity to express an interest in taking part by returning an expression of interest reply slip. If the person had capacity to consent, they returned the reply slip themselves or to a member of staff (NHS or third-sector organisation), who sent it on their behalf. If the person did not have capacity to consent, the reply slip was sent by a personal consultee. Those who responded were contacted by the researcher via their preferred method to discuss their participation.

Participants were asked to provide written consent. On arrival for the focus group or interview, the participants (and care partners) were met by a member of the research team, who went over information about the study and how the focus group or interview was going to run, and also went through the consent form with them confirming they had understood the information. They were given the opportunity to ask any questions and to have these answered. At this stage, the people living with dementia were asked to sign the consent form, with provision for verbal, recorded consent for those experiencing difficulty with writing. It was made clear from the start that a decision to take part in the study was entirely voluntary and that they could leave the study without an impact upon any care they receive. These processes provided adequate time to ensure that participants did not feel rushed or unable to ask questions at this point in their involvement.

##### Recruitment of care partners

We recruited a sample of care partners who met the following criteria:

- They were self-reported care partners (family member or friend) of a person with a diagnosis of dementia who met the study inclusion criteria described earlier.
- They spoke English.

We identified and recruited care partners who met the inclusion criteria using the following approaches:

- If their partner, a person living with dementia, had been recruited to the study.
- Care partners were also recruited via NHS trust clinics and third-sector organisations (Alzheimer’s Society or local memory cafes).

##### Recruitment process for care partners

Those care partners who met the study inclusion criteria were given information about the study and the opportunity to express an interest in taking part. If interested, they provided a preferred method of contact by returning an expression of interest reply slip. Those who responded were contacted by a researcher via their preferred method to discuss their participation. A study information sheet was given (either by post or in person by a researcher), highlighting that a focus group would be conducted with care partners for people living with dementia to gain insight into their perspectives on outcomes that are important for dementia care and services in the community to support people living with dementia at home. Care partners had opportunities to ask any questions and to discuss any aspects of the study with the researcher prior to their decision to participate. After providing the study information sheet (leaving at least 24 h before further contact), a member of the research team followed up potential participants to confirm their willingness to take part in the study.

When people confirmed their interest in taking part, invitations were sent to all participants seven days prior to the focus group to confirm date, time, venue and arrangements. Those who were not able to attend the focus group were asked to notify the researcher, and alternative arrangements made if they were still interested in the study.

Written consent was obtained from those willing to take part, before the focus group commenced and on the same date as the group. On arrival for the focus group, the participant was met by a member of the research team, who went over information about the study and how the focus group would proceed, and also went through the consent form confirming that they understood it. Any questions were answered. People were asked to sign the consent form. A copy of the consent form was stapled to the study information sheet and given to participants for their records. It was made clear from the start that their decision to take part in the study was entirely voluntary.

##### Recruitment of health and social care professionals and researchers

Health and social care professionals were recruited if they met the following criteria:

- Based in the UK and responsible for providing care and treatment to people living with dementia and their care partners, including general practitioners, consultants, nursing staff, allied health professionals, social care workers, and those who manage or run social community-based programmes
- UK researchers involved in dementia intervention studies involving people with dementia

Health and social care professionals and researchers were recruited through the following methods:

- Through our co-applicants (e.g. colleagues who lead and work on other work packages within the Neighbourhoods and Dementia study), networks (e.g. Neighbourhoods and Dementia Study Advisory Group and Scientific Advisory Groups, Academic Health Science Networks), study partners, established contacts working in the NHS and social care services, and academic research connections

##### Recruitment of policymakers

At UK national, regional and local levels, we approached and invited individuals from organisations involved in implementing policy and dementia care services, including clinical commissioners, joint commissioners and clinical leads with clinical commissioning groups, directors of public health, and local authorities. These contacts and networks were developed and supported by our study advisory group, scientific advisory group, clinical research network officers, Neighbourhoods and Dementia study colleagues, and academic research connections.

##### Recruitment process for health and social care professionals, researchers and policymakers

Potential participants were contacted via their preferred contact method as stated in the expression of interest slip, which was attached to a cover letter and a study information sheet. Upon receipt of acceptance, a mutually convenient date was arranged for the interview or focus group. An invitation letter was emailed to participants seven days prior to the interview or focus group to confirm date, time, venue and arrangements. All participants were asked to provide written consent prior to their participation.

#### Data analysis

The focus groups and interviews were audiotaped, fully transcribed verbatim, password protected and imported into NVivo Version 11. We used NVivo to store, manage and code all qualitative data. Full data analysis was not required in this study phase as the purpose of these qualitative data were for outcome identification. A coding framework was developed, drawn from qualitative studies with people living with dementia and the review of literature on existing outcomes reported as in studies and reviews (phase 1.2). Outcomes and potential outcome areas in the qualitative data were identified and coded in the framework, which constituted an extracted list of potential core outcomes [28]. All outcomes listed as important by people living with dementia will be included in the second phase of the study.

#### Phase 1.2: Literature review of existing outcomes reported in studies and reviews

The Cochrane Dementia and Cognitive Improvement Group of the Medical Sciences Division of Oxford University has created and manages a comprehensive and open access register of dementia studies. This database, known as the ALOIS database, is available online (http://www.medicine.ox.ac.uk/alois/). The register contains records of randomised controlled trials, controlled clinical trials and some other open-label studies. The advanced search function allows users to search by study aim, study design, intervention type or whether records are Cochrane studies.

To locate outcomes used in existing studies, the ALOIS database was searched for non-pharmacological interventions. At the time of searching, there were nearly 5000 registered studies in the ALOIS database. Of these, 1009 studies were identified as non-pharmacological and 248 studies evaluated a community-based intervention. The initial extraction scoping exercise revealed some duplication of outcomes across studies. Given the time constraints involved in undertaking the parallel qualitative element of the study (to ascertain outcomes important to key stakeholders, including people living with dementia), extracting outcomes from a sub-sample of the 248 studies was considered to be adequate. Thus, primary and secondary outcomes were extracted from a random 50% sample (*n* = 124). However, to be as exhaustive as possible, key reviews and qualitative studies (*n* = 8) and policy documents (*n* = 38) were also identified and outcomes extracted.

#### Phase 1.3: Bringing together the qualitative data and the data extracted from the literature: developing the long list

##### Researcher and clinician workshops

Outcomes extracted from phases 1.1 and 1.2 formed a long list of outcomes. To form an accessible Delphi survey, the outcomes needed to be grouped together where there were areas of commonality or duplication and mapped into outcome domains.

Reflecting previous work on COS development using a Delphi approach, the research team held eight meetings to remove areas of duplication, further consolidate areas of commonality and map outcomes in domains [29]. Participants in these meetings were from a range of health and social care research backgrounds, and included those with both clinical and caregiving experience.

Each meeting took between two and four hours and involved a series of discussions and exercises. Adapted from existing interactive focus group approaches [30, 31], every outcome was listed in a spreadsheet and printed on individual pieces of paper. These individual pieces of paper were placed onto a large table and were positioned during the discussion according to participants’ views on the rationale for mapping outcomes into domains, and merging or removing outcomes. Any disagreements were resolved through discussion.

At the end of the eighth meeting, it was universally agreed that this deliberative analysis was saturated.

### Phase 2: Delphi methodology

The Delphi method is a structured method for reaching consensus, in which participants complete sequential rounds of anonymised surveys. It is increasingly regarded as a robust approach to gaining consensus from different stakeholder groups [21]. This method, modified to ensure the participation of people living with dementia, will be used to reduce the range of items down to a COS.

In this study, two rounds of surveys will be distributed amongst each of the stakeholder groups. In round 1, participants will rate each outcome. There will also be an option for participants to add any additional outcomes and to provide a score for each outcome added. Round 2 involves participants reviewing the round 1 scores of other participant groups to reflect further on what is important. In round 2, participants will have the opportunity to change their response.

#### Delphi design: role of member involvement

Each outcome will have an associated statement and participants will be asked how important each statement is. The initial statements were designed by members of the research team. However, critical to the success of the Delphi approach involving the participation of people living with dementia is that research tools are accessible and understandable [32].

Recent work has highlighted the desirability of and the need to develop strategies and frameworks that consider the views of stakeholders in COS design [21, 33, 34]. Referred to as member involvement, we have consulted with people living with dementia and care partners to inform the design of COS research tools, including in the design of and how to engage people living with dementia appropriately in both rounds of the Delphi surveys. Individuals and groups who were involved in phase 1, and from other Neighbourhoods and Dementia study work programmes, were invited to consult. This was governed by the same practices and ethical procedures as the primary methods of data collection, and followed the recommendations and guidance for appropriate, respectful and safe inclusion of people living with dementia in research [35–37]. Member involvement consultation took place with three people living with dementia and five groups.

#### Recruitment of people living with dementia and care partners

People living with dementia who have capacity and care partners who participated in phase 1 will be approached and invited to participate in the Delphi survey. If they are interested in completing a Delphi survey, a researcher will contact them through their preferred method to provide information about the Delphi survey and to ensure that they understand the survey and have the opportunity to discuss any questions. Once they confirm their decision to take part in the Delphi survey, participants will receive an invitation to complete a researcher-supported survey or postal survey (round 1) and researcher-supported survey (round 2). A researcher-supported survey is where a researcher supports the participation of a person living with dementia in completing the survey, and is responsive to the preferences of the individual.

Additional people living with dementia and care partners who meet the study inclusion criteria will also be recruited from a variety of settings, which may include NHS trust clinics, primary care, third-sector organisations (e.g. Alzheimer’s Society) and the study website, and also via the study’s social media outlets, public engagement events within the local area and local newspaper articles, and from the Join Dementia Research register. We will recruit a sample of participants who meet the same criteria as phase 1. A list of these potential participants will be collected and stored in a password-protected database.

Those who meet the inclusion criteria will be given information about the Delphi survey and the opportunity to express an interest in taking part in the study. If potential participants are interested in the study, then they, or a member of staff on their behalf, will provide a preferred method of contact by returning an expression of interest reply slip. Those who respond will then be contacted by a researcher via their preferred method to discuss their participation. Potential participants will have the opportunity to ask any questions and discuss any aspects of the Delphi study with the researcher before making their decision.

#### Recruitment of health and social care professionals, researchers and policymakers

Health and social care professionals, researchers and policymakers participating in phase 1 will also be invited to take part in the Delphi study. Additional health and social care professionals, researchers and policymakers will also be identified and recruited through contacts and networks in the Neighbourhoods and Dementia study work programme, the clinical research network, social media, the study’s website, third-sector organisations and research networks (e.g. CHAIN; http://www.chain-network.org.uk), and attendees and presenters at relevant conferences and events.

Health and social care professionals, researchers and policymakers will be contacted by email directly to complete an online Delphi survey via an embedded link. Reminder emails will be sent. The importance of completing both rounds of the Delphi exercise will be clearly stated. If we find that particular groups are under-represented, we will target these specifically.

#### The Delphi survey scale

Although the Grading of Recommendations, Assessment, Development and Evaluation scale outlines the desirability of a nine-point scale (1–9) to rank the importance of each outcome [38], one of the few existing Delphi studies that did not make substantive modifications involving people living with dementia indicates that even a scale with as many as five points would be likely to be unsuccessful [32]. Nor is it advisable to use a scale that accommodates extremities and indicates that outcomes could be categorically unimportant. An example of this is using ‘strongly disagree’ (if written in the first person) or ‘not important’. For people living with dementia, it is unlikely that outcomes will categorically not be important [32]. It is preferable to ask participants to consider which areas are of less or greater importance, thus acknowledging that all outcomes have some importance.

This study will use a three-point scale that will not suggest categorical unimportance: (1) ‘not particularly important’, (2) ‘important’ and (3) ‘very important’. This three-point scale is a key modification to a regular Delphi process, for which preliminary member involvement has been received.

#### Delphi sample size

There is no consensus on the optimal sample size for a Delphi study [22] and it is common practice to use prior studies as an indicator of an appropriate sample size [39]. A recent review of Delphi COS studies suggests that the average number of participants for mixed-participant Delphi surveys such as this is 171 [21]. This Delphi survey will aim to purposively recruit approximately 200 participants. Of these, 20–30 will be people living with dementia. Given the potential that both rounds of the Delphi surveys will be researcher supported, this has resource implications (e.g. for researchers travelling to and from participants’ homes across the north-west of England). The other stakeholder groups will have approximately equal numbers of participants.

#### Demographic/other information

All participants who agree to take part in the survey will be registered with a unique identifier, which will be allocated to enable tracking of attributions at each round. Upon registration, participants will be asked to identify themselves with one or more of the stakeholder groups and provide basic demographic information.

For people living with dementia and care partners, this includes postcode, gender, ethnicity, religion, living arrangements, current employment status, length of diagnosis, and EQ-5D-3L measure (to provide a summary of health). For health and social care professionals, policymakers and researchers this includes ethnicity; age range; job title and role (and recent project involvement for researchers); organisation and location; qualifications and specialisms; how long they have been working in dementia care, services or research; other dementia-related positions held; and whether they have undertaken any dementia-specific training in the past three years.

#### Supporting participation of people living with dementia in both delphi rounds

##### Round 1: Postal or researcher-supported survey

In round 1, people living with dementia will have the choice of completing a postal survey or accessing researcher support to assist completion. However, round 2 will need to be completed with researcher support. There will be an opportunity at the end of the round 1 survey to add additional outcomes. For postal surveys, a free text comment box will be available. For surveys completed with researcher support, based on any conversations during the administration of the survey, the researcher will ask the participant if anything else is important to them and note anything down as an additional outcome.

##### Round 2: Researcher-supported survey and binary choice approach

The purpose of round 2 is to expose participants to the views of others, and ask them to reflect to attain areas of consensus. This involves presenting other participant groups’ feedback, typically in graphic form such as bar charts, pie charts and histograms. However, member involvement consultations with people living with dementia in this study have demonstrated how these feedback mechanisms tend not to be accessible, particularly for people with visuospatial impairments.

To include people living with dementia in round 2, researchers will initiate an interview format where participants will be reminded of their round 1 response. Along with a paper-based version of the survey, participants living with dementia will then be verbally presented with a single participant group response, namely that of health and social care professionals. This is based on the rationale that, from the wider COS literature and member involvement consultation in this study with people living with dementia, there tend to be differences of opinion between service users or patients and health and social care professionals [22].

There are three permutations to reviewing round 1 scores. First, where there is a clear difference between the two groups, this is likely to be phrased something like: ‘The last time you said xxx. Most health and social care professionals said xxx. Do you want to keep your answer or do you want to change your mind?’ Second, where there is more of an even split, this is likely to be phrased something along like: ‘The last time you said xxx. Health and social care professionals couldn’t make their mind up between xxx and xxx. Do you want to keep your answer or do you want to change your mind?’ Third, when the majority of health and social care professionals views reflect the person’s initial answer, this is likely to be phrased something like: ‘The last time you said xxx, and the majority of health and social care professionals said the same. Do you want to keep your answer or do you want to change your mind?’

This is another key modification to a more standard Delphi. This process will be administered by researchers in a calm and respectful manner. Care will be taken to emphasise that this does not mean the person with dementia’s initial answer was wrong and that, while there is no pressure for them to change their mind, they are being invited to reflect on their initial answer, and change their mind if they wish to.

##### Being responsive to individuals

Data collection considerations for both rounds will be responsive to the needs of individual participants. While we are offering the support of a researcher to go through the Delphi survey with participants (optional for round 1 and necessary for round 2), additional considerations might include the setting in which participants are most comfortable to complete the Delphi, either in their own home or in a familiar public place.

Another consideration is to provide the option for participants to complete either or both rounds in multiple sittings, or to allow people to only partially complete either round. While no set time limits have been placed on how long it will take for a person living with dementia to go through round 1 or round 2, we will judge what time is appropriate individually. Important to this is to recognise when participants are tiring. All the researchers and those who will offer researcher support have experience of communicating with people living with dementia.

Unlike other studies, aspects of inclusion and being responsive to the needs of people living with dementia mean that partial completion in this study will not be regarded as non-completion.

#### Randomising the order of the survey questions

The possibility that some people living with dementia may not be able or wish to complete all questions has implications for how the Delphi survey will be conducted. Under the usual Delphi process of presenting the survey items in the same set order to all participants, allowing partial completion carries a risk that items lower down in the list will receive substantially fewer, or possibly no, responses. To counter this, each participant will receive the items in an individually randomised order. In addition, over the time frame of data collection, sections or items with a lower number of responses will be identified and prioritised in subsequent randomised lists, where appropriate. This is an important consideration for accommodating participants living with dementia, and represents a further key modification to the Delphi method.

#### Gift as a recognition of participation

Participation in the Delphi survey involves completing two surveys. Specifically, for people living with dementia, who may complete one or both surveys in multiple sittings, it is possible that participation may involve an amount of time for which a recognition of involvement is appropriate. In this study, people living with dementia will be offered a £10 voucher for participation in each round. Participants from other stakeholder groups will be given a £10 voucher upon completion of round 2.

#### Analysis

The analysis protocol assumes that sufficient numbers of stakeholders from each group will respond.

##### Round 1

Data from the round 1 survey will be analysed separately for each stakeholder group. For each outcome, the number of participants who score the outcome and the distribution of rating scores will be summarised in a histogram. Additional outcomes listed by participants will be reviewed and checked by members of the research team to ensure they represent new outcomes. If there is uncertainty, the study advisory group will be consulted, and we will also draw on previous categorisations from outcome workshop materials. All outcomes will be carried forward to round 2.

##### Round 2

For each outcome, the number of participants who score the outcome and the distribution of rating scores will be summarised together with the number of participants who scored the outcome in both rounds. This process will be the subject of substantive member involvement with people living with dementia to ensure that their active participation is accommodated in round 2.

The responses of each stakeholder group will be analysed and compared within that stakeholder group, and the percentage agreement will be used to determine the focus of the final consensus meeting. Each outcome by stakeholder group will be classified as:

- Consensus in: 70% or more participants scored it as ‘very important’ and less than 15% of participants scored it as ‘not particularly important’.
- Consensus out: 70% or more participants scored it as ‘not particularly important’ and less than 15% of participants scored it as ‘very important’.
- No consensus: Anything else not included in the other two categories.

A list of the questions where ‘consensus in’ was met for one or more stakeholder groups will be presented and used in the following consensus meeting.

#### Consensus meeting

While there is no accepted definition of what constitutes consensus in relation to Delphi approaches [29], it is widely regarded as good practice to finalise consensus through one or more consensus meetings [21].

Consensus meeting approaches differ, with one key distinction being whether to host a single meeting with representatives from all stakeholder groups or to hold multiple meetings for representatives of each stakeholder group [40]. This study will convene a face-to-face consensus meeting with a sample of all Delphi study participants to discuss and attain agreement on the core outcomes. The meeting with be facilitated by a specialist independent chair. The meeting format and structure will be based on how best to accommodate the needs of each group, particularly people living with dementia. Though details will be finalised through research team meetings, existing consensus meeting approaches will be reviewed and member involvement consultation will be used to determine a desirable and workable approach. By the end of this meeting, we will have identified what outcomes to measure.

### Phase 3: Systematic reviews of outcome measurement instruments

Relating to the outcomes identified in the COS, we will identify, or where necessary conduct systematic reviews to identify and assess, the properties of existing outcome measures used in research for people living with dementia. Any systematic reviews undertaken will be registered on PROSPERO.

Measurement properties will be assessed using the Consensus-based Standards for the Selection of Health Measurement (COSMIN) checklist [41] and the COSMIN-COMET guidance. Measures will be assessed for published evidence of validity, reliability and responsiveness [42]. The COSMIN database of systematic reviews will be searched to check if previous researchers have done this already for a particular instrument of interest.

### Phase 4: Stated preference survey

A stated preference approach will be used to estimate the relative preferences of each stakeholder group for key outcomes (domains). A questionnaire (SP-CORE) will be developed from the results of phases 1–3 and refined by discussion with participants of the Delphi consensus meeting group and member involvement meetings. The latter are particularly important to ensure that the survey design enables people living with dementia to participate in the survey. Participants will be asked to take part in a pilot and the respondents for the pilot version of the SP-CORE will have the choice of completing a postal or electronic survey.

#### Recruitment

In earlier study phases, participants of each stakeholder group will be invited to complete the stated preference survey. If interest is expressed, a researcher will contact them through their preferred method. Information will be provided about the stated preference survey to ensure that they understand the survey and have the opportunity to discuss any questions. Again, a researcher-supported approach to the inclusion of people living with dementia in phase 4 will be developed in line with participation approaches from earlier study phases.

#### Analysis

The stated preference survey will use an orthogonal main effect design. Responses from the survey will be analysed using logistic or probit regression analyses as appropriate. The coefficients for each attribute will indicate the direction of preference for that attribute. Marginal rates of substitution will be calculated to estimate the relative utility of the attributes.

The elicited preference data will be used to explore the relative importance and preference for different outcomes included in the COS (domains), and to estimate preference weights that can be used to combine key domains into a single index [42, 43]. This index can be used to explore the cost-effectiveness of the development of a couple-orientated self-management intervention provided at home (part of another work programme [6] under the Neighbourhoods and Dementia study).

## Discussion

A COS represents the minimum outcomes for a research area. Studies of specific interventions or programmes will likely supplement the COS with relevant outcomes. Following on from other COS studies, this study has key strengths, including substantive qualitative work with stakeholders as part of a rigorous process of identifying outcomes, which the core set will be based on. The stated preference survey will provide information about the relative importance of the different outcomes. This will add to the information available for interpretation of the results of future evaluations for policy and practice.

It is important to acknowledge the sequential and innovative nature of this study. This study is the first, to the best of our knowledge, to be designed to enable the participation of people living with dementia. This is a key strength, made possible by a modified Delphi process. It was necessary that the design of the modified Delphi process be influenced by phase 1.1 (qualitative data collection) and the member involvement consultation in the early part of phase 2. This has resulted in a Delphi study design responsive enough to accommodate the participation of people living with dementia.

The first modification to the Delphi study design is the use of a three-point scale, instead of the regular nine-point scale. The second modification is to complete surveys with people living with dementia in an interview-like format. The third modification is to only expose people living with dementia to the results of health and social care professionals (as opposed to all stakeholder groups) in the second round of the Delphi. The fourth modification is to randomise Delphi questions for people living with dementia according to the completion rates of all Delphi questions. All four modifications are key strengths of this work and are important developments in involving people living with dementia in research in a discipline and field where previous attempts may have been less thoroughly done, if at all.

Some potential limitations should also be highlighted. Firstly, data collection has been and is limited to the UK. Furthermore, given the resources and the need for researcher-supported data collection, the sample of people living with dementia is mostly restricted to the north-west of England (although participants have been, and will be, from diverse parts of this region). The importance of outcomes may, of course, vary within and across cultures as well as within and across the perspective of the different stakeholder groups [38]. It is likely that further work will be needed when developing outcomes and statements for an international audience.

Secondly, the use of an online survey tool for all stakeholders (except people living with dementia) will limit participation to those who are computer literate. Thirdly, invariably the Delphi surveys will tend to be completed by people with mild to moderate dementia. While we will not gauge the views of people with advanced dementia in phases 2 and 3, these were gained in phase 1. Despite this particular limitation, as stated above, this study does mark progress in the participation in research of people living with dementia.

This study design and the Delphi method are increasingly being recognised as a robust approach to forming COSs [20]. This study will capture the views of people living with dementia, who, along with the other stakeholders, will come to a consensus around what outcomes should be measured in relation to non-pharmacological and community-based programmes. In doing so, this study will move closer to providing researchers with outcomes that are important to people living with dementia and other key stakeholders, thereby increasing the comparability of studies evaluating interventions and reducing reporting bias (Additional file 1).

### Trial status

Recruitment to phase 1 and member involvement that informed the design of phase 2 has been completed. At the time of submission, phase 2, involving the Delphi survey, is due to start in autumn 2017. The consensus meeting will take place shortly after the second round of the Delphi survey. Recruitment for phase 4 will begin in the middle part of 2018.

## Additional file

Additional file 1: Recommended items to address in a clinical trial protocol and related documents*. (DOC 119 kb)

## Abbreviations

COMET: Core Outcome Measures in Effectiveness Trials
COS: Core outcome set
COSMIN: Consensus-based Standards for the selection of health Measurement Instruments
ESRC: Economic and Social Research Council
NHS: National Health Service
NIHR: National Institute for Health Research

## Footnotes

1 Care partners is the term selected by involvement groups in work programme 1 of the Neighbourhoods and Dementia study to refer to those who look after, support and care for someone living with dementia, in a non-professional unpaid capacity. This may be a family member, friend or neighbour.
